# Extramedullary haematopoiesis in patients with transfusion dependent β-thalassaemia (TDT): a systematic review

**DOI:** 10.1080/07853890.2022.2048065

**Published:** 2022-03-09

**Authors:** Eihab A. Subahi, Fateen Ata, Hassan Choudry, Phool Iqbal, Mousa A. AlHiyari, Ashraf T. Soliman, Vincenzo De Sanctis, Mohamed A. Yassin

**Affiliations:** a Department of Internal Medicine, Hamad General Hospital, Hamad Medical Corporation, Doha, Qatar; b Department of Internal Medicine, Faisalabad Medical University, Faisalabad, Pakistan; c Critical Care Unit, Hamad General Hospital, Hamad Medical Corporation, Doha, Qatar; d Pediatrics and Endocrinology Department of Pediatrics, Hamad Medical Center, Doha, Qatar; e Department of Pediatrics, University of Alexandria, Alexandria, Egypt; f Pediatric and Adolescent Outpatient Clinic, Quisisana Hospital, Ferrara, Italy; g Department of Medical Oncology/Hematology, National Centre for Cancer Care and Research, Hamad Medical Corporation, Doha, Qatar

**Keywords:** Thalassaemia, transfusion-dependent thalassaemia, extramedullary haematopoiesis

## Abstract

**Introduction:**

Around 5% of the world’s population is expected to have some degree and type of thalassaemia. Beta thalassaemia (BT) occurs due to a deficient production of the beta-globin chain of haemoglobin. Extramedullary haematopoiesis (EMH) is one of the complications of BT, mainly observed in minor/intermedia subtypes. EMH is the production of blood cells outside the marrow as a compensatory response to longstanding hypoxia. Due to chronic transfusions, it is not expected in patients with beta-thalassaemia major (BTM). However, there are increasingly reported cases of EMH in BTM. The incidence of EMH in BTM is thought to be <1%. We aim to pool the available data and provide cumulative evidence on the occurrence of EMH in BTM patients.

**Methods:**

This is a systematic review of case reports, series, and retrospective studies that presented data on the occurrence of EMH in BTM patients. Data were recorded and analyzed in Microsoft Excel 2016 and SPSS 26. The protocol has been registered in PROSPERO: CRD42021242943.

**Results:**

Data from 253 cases of EMH in BTM patients were extracted with a mean age of 35.3 years. Mean haemoglobin at presentation with EMH was 8.2 mg/dL. Lower limb weakness was the most common presenting feature (*N* = 23) (paraspinal EMH). Magnetic resonance imaging (MRI) was the most widely used diagnostic modality (226). Overall, blood transfusion was the commonest reported treatment (30), followed by radiotherapy (20), surgery (15), hydroxyurea (12), steroids (6), and exchange transfusion (2). An outcome was reported in 20% of patients, all recovered, except one who died as a result of nosocomial infection.

**Conclusion:**

EMH is rare in BTM and can occur in any organ system with varied clinical features. MRI can effectively diagnose EMH, and conservative management has similar results compared to invasive treatments. Larger studies, focussing on outcomes may enhance guidelines on preventive and therapeutic strategies for managing EMH in BTM.

**KEY MESSAGES**
- Extramedullary haematopoiesis is a rare complication in beta thalassaemia. Although it is more common in non-transfusion dependent thalassaemia, increasingly reported cases suggest a higher prevalence of EMH in TDT than what is known before.
- There are no clear guidelines on the management of EMH in TDT, with reported patients showing similar outcomes with conservative invasive treatment modalities.
- More extensive and preferably prospectively designed studies are required focussing on the management of EMH and its outcomes in patients with TDT to formulate evidence-based guidelines.

## Introduction

Hemoglobinopathies are the most common monogenic diseases worldwide, characterized by autosomal recessive inherited defects affecting the synthesis of normal haemoglobin, mainly HbA. Approximately 1–5% of the global population are carriers of a genetic thalassaemia mutation. β-thalassemias can be subdivided into two main types depending on the clinical features: Thalassaemia Major (TM) patients, who are transfusion-dependent thalassaemia (TDT), and Thalassaemia Intermedia (TI) patients, who are non-transfusion-dependent thalassaemia (NTDT). TDT refers to the group of patients who require regular blood transfusions for survival from early life, including severe forms of haematological phenotypes of β thalassaemia, e.g. homozygous β0 thalassaemia, β0/β+, β/δβ, and others. Regular red blood cell (RBC) transfusions improve anaemia, compensatory bone marrow expansion, bone changes, and splenomegaly, restore physiological growth throughout childhood and ensure survival. The most serious disadvantage of life-saving transfusions is the inevitable transfusion-related iron input and organ deposition [1].

The majority of the complications of TDT are related to iron overload and bone-deforming marrow expansion with extramedullary haematopoiesis (EMH) [2–8]. EMH involves the development and advancement of blood cells in locations other than the long bones, such as the pelvis, spine, and sternum, outside of the bone marrow cavity. EMH is a compensatory response to poor bone marrow function, which can result in the production of ectopic haematopoietic components outside of the bone marrow and peripheral circulation. EMH aspires to resemble natural bone marrow. It is more prevalent in individuals with thalassaemia intermedia who are not transfused, and it is less common in poorly transfused β-TM patients whose erythropoiesis is not properly controlled by transfusions [9]. However, it is also present in other haematological diseases, such as myelofibrosis, polycythaemia vera, leukaemia, lymphoma, and sickle cell anaemia. EMH can be seen in upto 20% of NTDT patients compared to those with TDT, where the incidence is <1% [10]. EMH has been reported in nearly every organ but is frequently seen in hepatosplenic areas, potentially producing foetal haemoglobin.

Non-hepatosplenic EMH is seen in various anatomical locations including but not limited to the lungs, gastrointestinal tract, urinary tract, adrenal glands, prostate, peritoneum, skin, breast, central nervous system, and paravertebral areas. The most common site involved by EMH in thalassaemia patients is the spinal column, particularly the thoracic region. The reason for the increased frequency of EMH around the spinal column, and more specifically at the thoracic levels, is unknown. However, early diagnosis of EMH as an epidural or paraspinal lesion is important since chemotherapy and radiotherapy are effective therapeutic options in most patients who suffer few if any, symptoms.

The diagnosis and treatment of EMH are critical for avoiding cord compression and lasting neurological abnormalities, as well as lowering the risk of irreparable neurologic damage in paraspinal regions. Treatment options are controversial and specific treatment may not be required unless symptoms accompany EMH. Treatment options for thalassaemia patients with EMH depend on the location and mass effect symptom, including hyper transfusion, surgical excision, radiotherapy, hydroxyurea, or various combinations therapy [10,11]. The main aim of this review is to pool all the available data on the presentations and management of EMH in the context of TDT, providing summary statistics.

## Patients and methods

### Literature search

A systematic literature search was performed for articles using PubMed, Google Scholar, and Scopus for any date up to December 2020, for all the articles describing EMH in the context of TDT, following the Preferred Reporting Items for Systematic reviews and Meta-Analyses (PRISMA) guidelines for systematic reviews [12]. All of the articles in English were analyzed by two authors individually. Our predefined search term comprised of patients with transfusion-dependent thalassaemia who developed extramedullary haematopoiesis. The following search term was used: “beta-thalassemia major” OR “transfusion-dependent thalassemia” OR “TDT” and “Extramedullary hematopoiesis” OR “non-medullary hematopoiesis”. The extracted articles were screened by title and abstract, following an in-depth screening of the relevant studies.

### Study selection

**Figure 1.**
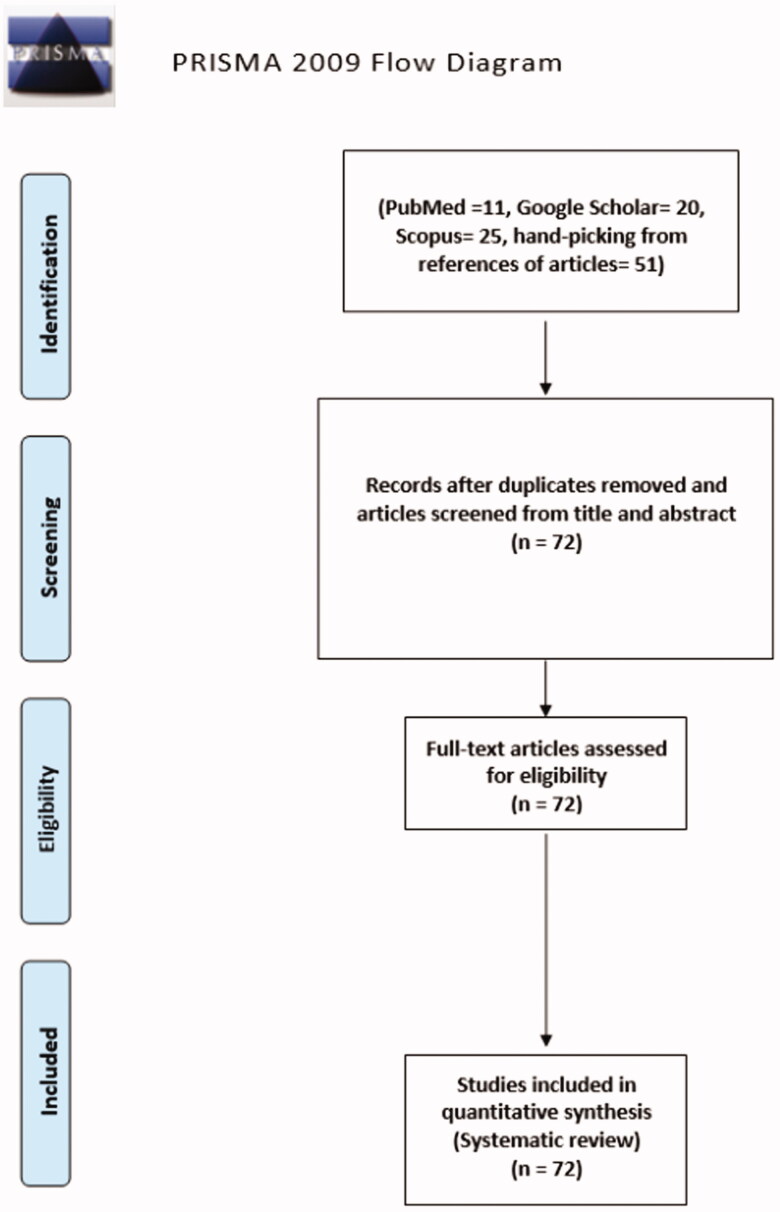
Prisma flowchart with details of the article screening process.

Our inclusion criteria included all articles in the English language describing patients (pediatric and adult) who had a confirmed diagnosis of beta thalassaemia major and developed EMH, confirmed radiologically and/or *via* biopsy. Articles that did not present new data were excluded from our review. Any patient with EMH but non-transfusion dependent were excluded from the study. Additionally, patients with TDT but an unconfirmed diagnosis of EMH were also excluded. Eligible studies (*N* = 72) reported the EMH in TDT and included case reports, case series, and retrospective studies (Figure 1). The quality of the added cases was analyzed by two reviewers independently (FA and ES) using the Joanna Briggs Institute case report appraisal checklist for inclusion in systematic reviews [13]. In case of disagreements in quality assessment and article screening, a third reviewer (MAY) independently analyzed the disputed articles to conclude.

### Data collection

Epidemiological parameters, clinical findings, laboratory investigations, sites of EMT, treatments are given, and outcomes were noted in all the cases where available. Data were recorded and analyzed in Microsoft Excel 2016 and SPSS 26 using summary statistics (means/medians, and numbers with percentages). Data was collected by Four reviewers independently (FA, ES, PI, and MA).

## Results

A total of 253 cases of EMH were reported in patients with BTM. Our literature review identified two retrospective studies (one with 167 cases and one with 15 cases) and 70 case reports. Table 1 summarizes the demographic and clinical findings among the 253 patients.

**Table 1.** Demographic characteristics of BTM patients with EMH.

Characteristics	Results (*N* = 253)
Gender	
Males	144 (56.9%)
Females	103 (40.7%)
Not reported	6 (2.3%)
Mean age at presentation of EMH (years)	35.3 ± 0.5
Ethnicity (*N*, %)	
Not reported	64 (25.6%)
Italian	167 (66%)
Iranian	7 (2.8%)
Greek, Chinese, African	2 (0.8%)
Pakistani, Palestinian, Emirati, Iraqi, Qatari, Liberian, Australian, Mediterranean, Cambodian	2 (0.8%)
	2 (0.8%)
	1 (0.4%)
Mean haemoglobin (mg/dL)	8.2 ± 2.1
Site of EMH (*N*, %)	
Not reported	3 (1.2%)
Around the Spinal cord	222 (87.7%)
Skull	6 (2.4%)
Spinal cord	5 (1.9%)
Intracranial	4 (1.6%)
Thorax	3 (1.2%)
Spleen	1 (0.4%)
Liver	2 (0.8%)
Mediastinum	1 (0.4%)
Adrenal gland	6 (2.4%)
Diagnostic tests (*N*, %)	
Not reported	4 (1.6%)
MRI	226 (89.3%)
CT Scan	25 (9.9%)
Biopsy	9 (3.6%)
PET scan	4 (1.6%)
SPECT	3 (1.2%)
Ultrasound	3 (1.2%)
Myelogram	1 (0.4%)
X-ray	1(0.1%)
Treatment (*N*, %)	
Not available	174 (68.8%)
Blood transfusions to increase pre-transfusion Hb	30 (11.8%)
Exchange transfusion	2 (0.8%)
Hydroxyurea	12 (4.7%)
Steroids	6 (2.4%)
Radiotherapy	20 (7.9%)
Surgery	15 (5.9%)
Outcome	
Not reported	201 (79.4%)
Recovered	52 (20.5%)

The mean age at presentation with EMH was 35.3 ± 0.5 years. Fifty-seven (57.7%) were males. Most of the reported cases were from Italy (66%), followed by Iran (2.8%). Ethnic background was not reported in 26% of patients. The mean haemoglobin (Hb) at presentation was 8.2 ± 2.1 mg/dL. The involved sites of EMH were around the spinal cord (*N* = 222, 87.7%) followed by the skull (*N* = 6, 2.4%), inside the spinal canal (*N* = 5, 1.97%), and intracranial (*N* = 4, 1.6%). Adrenal glands were involved in six patients (2.4%). Other rare sites of EMH included thorax (*N* = 3, 1.2%), mediastinum (*N* = 1, 0.4%), liver (*N* = 2, 0.8%), and spleen (*N* = 1, 0.4%), and the site of EMH was not mentioned in three patients (1.2%).

**Figure 2.**
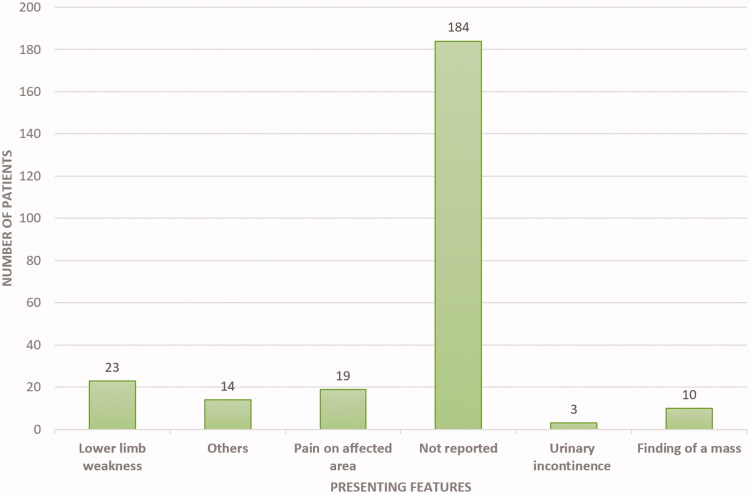
Frequency of reported presenting features of EMH in BTM patients.

Presenting features were not specified in a majority (*N* = 184, 72%) of cases (including the two retrospective studies) [14,15]. In the remaining patients, the authors reported a spectrum of presentation features. Thirty-two patients presented with clinical features of spinal cord compression. We found a small number of patients with symptoms of compression compared to the total number of patients with EMH around the spine. This is mainly because, in the two retrospective studies, which had 182 patients, the patients did not have any symptoms of EMH [14,15]. Lower limb weakness was reported in 23 (9.0%) patients. Nineteen (7.5%) patients presented with local pain at the site of EMH, whereas 3 (1.18%) presented with urinary incontinence. Ten patients (4%) presented with the finding of mass, either visible or incidental, on examination or imaging (Figure 2).

Various diagnostic modalities were used, either alone or in combination, to identify the EMH. Magnetic resonance imaging (MRI) was the most commonly used diagnostic method (*N* = 226, 89.3%), followed by computed tomography (CT) scans (*N* = 25, 9.9%). A histopathological diagnosis was made in only nine patients (3.6%). Other rare modalities used to identify EMH included positron emission tomography (PET) scan (*N* = 4, 1.6%), single-photon emission computerized tomography (SPECT) scan (*N* = 3, 1.2%), ultrasound (*N* = 3, 1.2%), myelogram (*N* = 1, 0.4%) and X-ray (*N* = 1, 0.4%).

Not all patients required therapy and/or treatment was not described in 174 (68.8%) patients. Although therapy and/or treatment were not required for all patients, they were reported in 85 (33.5%) patients. The treatments were given either alone or in combination. Blood transfusion (BT) to increase the pre-transfusion Hb level was the commonest reported treatment (*N* = 30, 11.8%), followed by radiation therapy (RT) (*N* = 20, 7.9%), surgery (*N* = 15, 5.9%), Hydroxyurea (HU) (*N* = 12, 4.7%), steroids (*N* = 6, 2.4%), and ET (*N* = 2, 0.8%) (Table 1).

An outcome was described in only 52 patients (20.5%). Among these patients, all recovered without any mortality, except one patient. The patient who died was an 11-years old female who had TDT and developed EMH as a homogeneous opacity of the left hemithorax. The patient developed hemothorax after thoracocentesis was performed, and eventually died as a consequence of nosocomial infection [16]. Table 2 summarizes the treatment lines in these patients with reported outcomes.

**Table 2.** Treatment lines among the patients with a reported outcome.

Treatment	Count (*N* = 52)
In various combination	
Blood transfusion	27 (51.9%)
Exchange transfusion	1 (1.9%)
Hydroxyurea	11 (21.1%)
Steroids	6 (11.5%)
Radiotherapy	19 (36.5%)
Surgery	12 (23%)
Exclusive treatment	
Blood transfusion	6 (%)
Hydroxyurea	1 (%)
Steroids	1 (%)
Radiotherapy	5 (%)
Surgery	12 (%)

## Discussion

**Figure 3.**
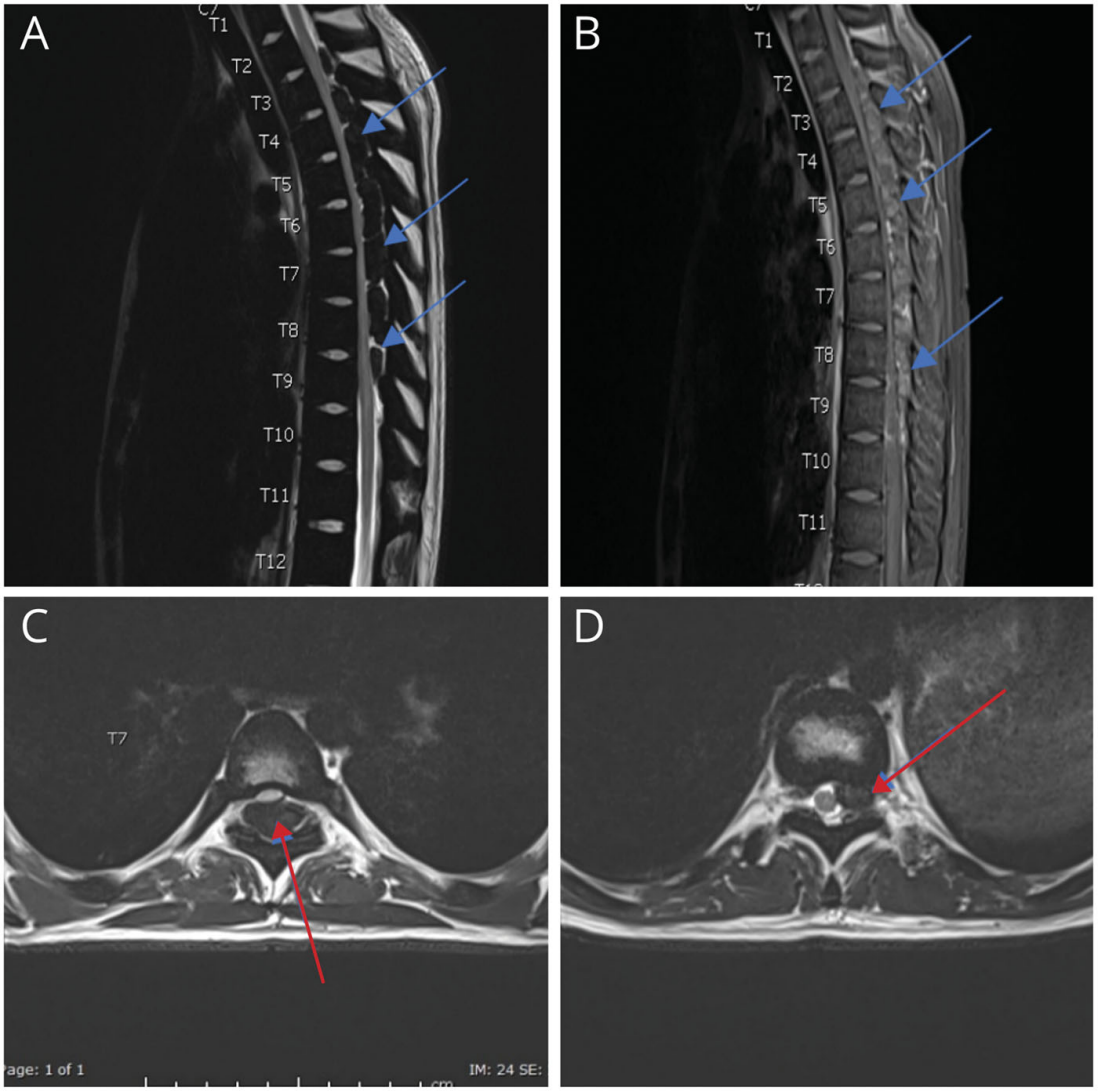
MRI spine: intraspinal epidural extramedullary masses T2 (lowered border) to T9 (arrows in A and B). Anterior displacement and compression of the cord in the thoracic spine is secondary to the compression by the EMH (arrow in C at T7 level) reaching upto the left neural foramen (arrow in D at T10 level). *Note*. Image taken from Clinical Case reports from a previously published article [18]. The licence of the article (CC-BY) permits unrestricted reuse of the published work.

**Figure 4.**
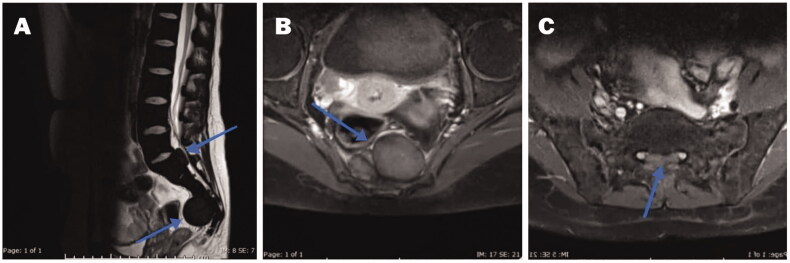
MRI spine: (A) Sagittal T2 WI showing a diffuse low-signal intensity of the bone marrow of a well-defined isointense mass (5.3 × 3 cm) in the presacral region (blue arrow). A second mass (2 × 1 cm) in anterior extradural space posterior to S1 level (Blue arrow). (B) Mass enhancement seen in the axial postcontrast fat-saturated image at the presacral area (Blue arrow). (C) Axial postcontrast fat-saturated image at S1 level showing anterior extradural mass with slightly high signal intensity (blue arrow) at the midline, with displacement of the S1 nerve roots laterally. *Note*. Image taken from Clinical Case reports from a previously published article [42]. The licence of the article (CC-BY) permits unrestricted reuse of the published work.

**Figure 5.**
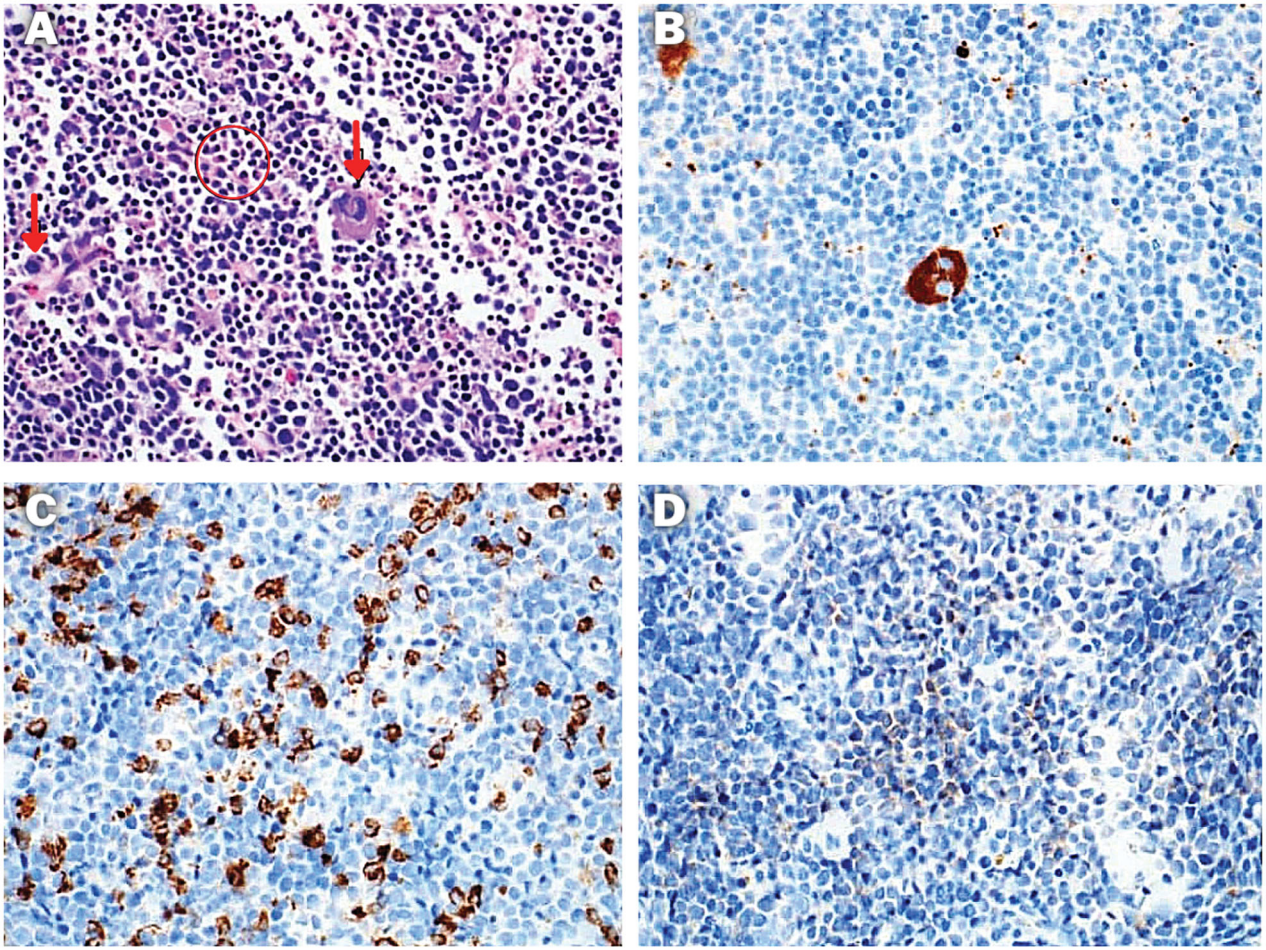
(A) Haematoxylin and eosin-stained slide showing lymphoid tissue comprising tiny lymphocytes with scattered megakaryocytes (arrows), and clusters of myeloid and erythroid cells (circled). (B) Immunohistochemical stain “CD61” highlighting the cytoplasm of megakaryocyte in brown chromogen. (C) Immunohistochemical stain “MPO”, highlighting the myeloid precursors. (D) Immunohistochemical stain “Hemoglobin A”, highlighting the erythroid precursors. *Note*. Image taken from Clinical Case reports from a previously published article [42]. The licence of the article (CC-BY) permits unrestricted reuse of the published work.

Thalassaemia is one of the commonest encountered hemoglobinopathies, with an annual addition of 40,000 new cases and more than 80 million carriers [17]. EMH occurs in various bone marrow disorders and is most seen in thalassaemia. This review illustrates an updated comprehensive review about EMH in TDT in comparison to other non-transfusion-dependent thalassemias (NTDT). The main idea that inspired us to conduct the review was our experience with two such cases in Qatar’s National Centre for Cancer Care and Research. The first patient was an 18-year-old male known to have TDT and regularly followed with BT every three weeks. The patient did not have any symptoms of anaemia, and the haemoglobin was above 8 mg/dL at presentation. However, he presented with lower back pain and a spastic gait. EMH was diagnosed on MRI (Figure 3). As there are no guidelines on the management of EMH, invasive non-invasive methods were contemplated, and the patient eventually underwent RT with a good outcome [18]. The second case was 31 years old female with TDT who presented with low back pain. Her Hb at presentation was 9.8 mg/dl. EMH was diagnosed as extradural masses on MRI (Figure 4). However, a confirmation was made *via* biopsy, which revealed erythroid, megakaryocytes, and myeloid precursors (Figure 5) [19]. The patient eventually underwent surgery with a resolution of her presenting features. Up to date, there are no clear guidelines for the best method/s of diagnosis or management (medical, radiological surgical, and invasive or non-invasive). These symptomatic patients with EMH require scientific attention for appropriate and timed management of complication/s associated with EMH.

Extraosseous and paraosseous EMH are the two forms of EMH [20]. Extraosseous haematopoiesis occurs in organs containing multipotent stem cells, such as the spleen and liver, and produces haematopoietic foci that are not associated with bone marrow. This is evident in diseases like myelofibrosis and thalassaemia, which hinder the marrow from producing enough RBCs. Splenomegaly or hepatomegaly, which can present as early satiety, bloating, pressure, or stomach discomfort, is common in patients. Para osseous EMH, in contrast to extraosseous EMH, arises surrounding the bone as a result of hyperactive marrow herniation [20]. It is more evidently seen in Sickle cell anaemia and thalassaemia as the erythroid marrow turnover is faster. Clinically, para osseous EMH may go unnoticed until enough cells form a tumor-like mass that causes symptoms [21].

**Figure 6.**
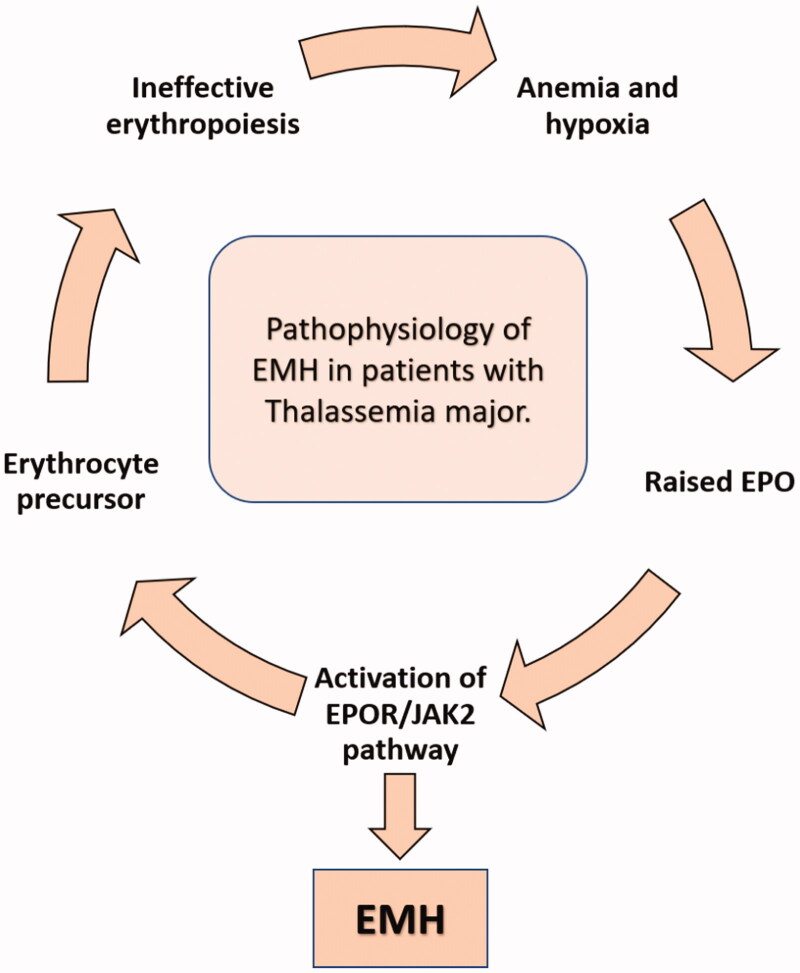
The pathophysiology of EMH in BTM. Concept reused with permission from Yang et al. [22].

The main pathophysiology of EMH lies in the mechanism of regulatory feedback in the erythropoietic cells. Although patients with TDT are regularly transfused, there remains subclinical anaemia as Hb levels are not targeted to be in the normal range. The chronic anaemic state creates hypoxic signals, resulting in increased erythropoietin production (EPO). This rise in the level of EPO activates the EPO receptor Janus kinase-2 pathway (EPOR-JAK2). The primary end result of this pathway is intended to be an increased level of erythrocyte production from the erythrocyte precursors. However, in cases where there is ineffective erythropoiesis, EMH occurs as a compensatory response to the increased signalling (Figure 6) [22]. In addition to the well-known and apparent complications of EMH due to the mass-effect and anatomic sites involved, recent data links EMH to a proatherogenic state. The haematopoietic stem and progenitor cells (which are the precursors of erythroid cells leading to EMH) also produce monocytes and other circulating inflammatory cells, which result in the augmentation of atherosclerosis. The process of EMH also alters the metabolism of lipids and cholesterol-efflux pathways, ultimately resulting in atherogenesis [23]. Given the well-known disease burden secondary to atherosclerosis and its complications, it is vital to further understand this link between EMH and atherogenesis further.

EMH usually regresses or disappears after treatment with different modalities, i.e. blood transfusions, steroids, and hydroxyurea. However, RT or surgical intervention can be required in some clinical symptoms requiring immediate intervention. In this review, we found that the highest number of EMH in TDT is reported from Italy. The MIOT network (an Italian cooperative group of nine MRI sites and 70 thalassaemia centers) conducted one study in 2015 [14]. EMH was detected in 167 patients with BTM. The mean age was 38.93 ± 6.10. This is comparable to the mean age reported in our review (35.3 years). In our review, 144 patients (56.9%) were males. Similarly, Ricchi *et al.* reported EMH in 91 males (54.4%) of their patients with no statistical difference between males and females [14]. The differences in mean ages and gender distributions can be attributed to the different study designs and patient populations. However, it can be concluded that EMH in BTM presents mainly in adulthood.

These findings suggest that the demographics of EMH in TDT are distinct compared to those with NTDT. In BT intermedia, studies have reported a male to female ratio of up to 5:1 [24]. The mean age of presentation with EMH in NTDT was lower than TDT. Yumei Huang *et al.* reported a mean age of 27.5 years [25]. EMH is related to chronic and prolonged anaemia that leads to ineffective erythropoiesis. In TDT, patients’ repeated-timed transfusion therapy reduces erythroid marrow stimulation and expansion, preventing and/or delaying the occurrence of EMH in BTM compared to NTDT.

A previous study found a mean pre-transfusion Hb of 9.63 ± 0.91 g/dL at the presentation in TDT patients presenting EMH compared to the pre-transfusion Hb of 8.2 ± 2.1 g/dL in our review [14]. In our review, the mean pre-transfusion Hb was lower than the minimum pre-transfusion recommended Hb of 9 g/dL. The mean Hb levels among TDT patients on transfusion were similar to those with NTDT and EMH [25]. Similarly, data from NTDT with EMH confirmed that the thoracic and dorsal regions were the most frequent localizations (98.8%). Our review found that the spinal and paraspinal involvement occurred in 89.7% of patients with TDT and EMH. However, our review revealed other rare sites of EMH in TDT. These include the skull, adrenal glands, liver, lungs, and parotid glands [26–31]. It is important for the physicians to be aware of these rare involvements to make a correct diagnosis timely. Furthermore, the modality of diagnosis and the management strategies may depend on the site involved.

Our review showed that EMH in TDT patients could be diagnosed incidentally without symptomatology. In one study, 15 patients were diagnosed with EMH incidentally while having scans for other reasons [15]. On the other hand, symptomatic patients presented with variable complaints. Presentation of these patients relied mainly on the anatomic site involved [25]. The commonest presentation reported was lower limb weakness. Other presentations included headaches, hearing impairment, vision loss, nocturia, tooth decay, and chest pain [27,32–36]. These presentations appeared quite atypical when considering EMH; hence there is a high chance of missing the diagnosis. Therefore, physicians shall be aware of these atypical presentations in thalassemic patients.

The optimum pre-transfusion Hb levels for TDT recommended by Thalassaemia International Federation are at least 9 g/dL in non-cardiac patients, whereas a higher minimum level (11 g/dL) in patients with cardiac complications. A lower pre-transfusion Hb than recommended can lead to complications of TDT related to under transfusion (mainly EMH), whereas maintaining a higher than recommended Hb can lead to transfusion-related complications, such as iron overload, osteoporosis, and pulmonary hypertension [37]. Studies have shown that despite clear guidelines, only a small number of TDT patients maintain the recommended pre-transfusion Hb levels. This percentage was as low as 38% in one study [38]. This might be due to more established over transfusion complications compared to less explored under transfusion complications, including EMH. Blood markers (such as nucleated red blood cells or soluble transferrin receptors) should be recorded before transfusions in TDT patients to assess their pre-transfusion states. Index of Ineffective Erythropoiesis (plasma concentration of soluble transferrin receptor divided by the absolute reticulocyte count) is a new and probably more accurate marker of the pre-transfusion state in such patients which is proposed and not yet validated [39]. Although in the added studies in this review, the authors did not present the parameters which could be used to assess the optimal pre-transfusion states, a mean Hb level of 8.2 ± 2.1 g/dL suggests that the majority of patients were not in an optimum pre-transfusion state which was required to avoid complications, such as EMH. More studies on EMH in TDT can help identify its true prevalence and outcomes so that the treating physicians can be more cautious in maintaining an optimum (rather than under-transfused) state in their TDT patients.

To our knowledge, there is no evidence-based regimen that recommends the best treatment approach to patients with EMH in TDT. Potential management options included hydroxyurea, blood transfusion, steroid, radiotherapy, surgical decompression, or a combination of these modalities. In most of the added cases, the reasons to choose one therapy over the other were not clearly mentioned. However, generally conservative management (blood transfusion to overcome the under-transfused state which caused the EMH) has opted in patients with minor or no symptoms. In contrast, more aggressive and curative approaches (steroids, cytoreductive therapies, radiotherapy, surgery) were used in cases with severe or debilitating symptoms or critical sites of EMH, such as paraspinal with neurological compromise. If the patients are in an under-transfused state (especially without any manifestations of iron overload), long-term management should focus on optimizing pre-transfusion Hb levels to >9 g/dL [40]. The rest of the management should be individualized based on the EMH location and presentation. As the haematopoietic tissue is susceptible to radiation, low-dose radiation is a potentially curative treatment option for EMH [41]. Patients who cannot receive blood transfusions or where radiotherapy seems ineffective may benefit from cytoreductive therapy, such as HU. Usually, surgical removal of EMH mass is the last resort generally or maybe the first line only in patients presenting with alarm signs, such as spinal cord compression with neurological manifestations.

One study recommended HU as the first line of management for EMH in BT patients [28]. All of the treatments mentioned above have been tried, either alone or in combination with each other. Choosing the management lines mainly relies on the site of involvement, the severity of symptoms, and physician and centre expertise. We could not find any study comparing different therapeutic approaches and their outcomes in BTM patients who developed EMH. Our review found similar results (wherever outcomes were reported) with conservative and invasive methods. Therefore, it is imperative to study the effects of the treatment options (non-invasive invasive) prospectively so physicians may have clear guidelines based on the best treatment options. If the invasive methods, such as RT or surgical removal show no better results compared to non-invasive methods (blood transfusion and HU), patients can avoid surgery and RT complications. However, this question can only be answered with more clear therapeutic data from well-designed prospective studies. Our data showed that surgery was commonly used as an exclusive treatment in 12 of the EMH cases; however, a good outcome was reported following both surgical approach and other conservative tactics.

The main strength of our review lies in the fact that it is its extensive revision of data on TDT patients who had EMH. This review shows that although EMH is not as common in TDT compared to NTDT, it can represent a significant medical problem that requires attention and proper management. However, there are limitations of our study as well. We could not perform statistical analysis on outcomes based on various treatment modalities used mainly because of a lack of outcomes in the studies. Unfortunately, outcomes were not reported in the two retrospective studies and some case reports. In addition, a lot of data (*N* = 70) was derived from case reports and series, which may affect the external validity of the review.

## Conclusion

Although rare, EMH may occur in patients with TDT and lead to significant clinical problems. The age of presentation appears to be higher than seen in NTDT. The most common site of EMH in TDT patients is around the spine. However, EMH can affect various organ systems. Both conservative and invasive treatment lines are being used to manage EMH in TDT patients, with no fair comparison of their outcome. As EMH is increasingly observed in TDT patients, it is imperative to study this phenomenon extensively, focussing on management and outcomes to find out guidelines for their management. Treating physicians should aim to attain the recommended pre-transfusion levels of Hb to at least 9 g/dL to avoid EMH. Treating physicians should also actively look for EMH in TDT patients, especially those unable to achieve the minimum recommended pre-transfusion Hb levels, to catch and manage EMH early and effectively with an individualized treatment plan.

## Supplementary Material

Supplemental Material

## References

[1] De Sanctis V, Kattamis C, Canatan D, et al. β-Thalassemia distribution in the old world: an ancient disease seen from a historical standpoint. Mediterr J Hematol Infect Dis. 2017;9(1):e2017018. 28293406 10.4084/MJHID.2017.018 PMC5333734

[2] Soliman A, Yassin M, Al Yafei F, et al. Longitudinal study on liver functions in patients with thalassemia major before and after deferasirox (DFX) therapy. Mediterr J Hematol Infect Dis. 2014;6(1):e2014025. 24803998 10.4084/MJHID.2014.025 PMC4010606

[3] Yassin MA, Soliman AT, De Sanctis V, et al. Effects of the anti-receptor activator of nuclear factor kappa B ligand denusomab on beta thalassemia major-induced osteoporosis. Indian J Endocr Metab. 2014;18(4):546–551. 10.4103/2230-8210.137516 PMC4138914 25143915

[4] Soliman A, Yasin M, El-Awwa A, et al. Acute effects of blood transfusion on pituitary gonadal axis and sperm parameters in adolescents and young men with thalassemia major: a pilot study. Fertil Steril. 2012;98(3):638–643. 22749224 10.1016/j.fertnstert.2012.05.047

[5] Soliman AT, Yassin M, Majuid NM, et al. Cortisol response to low dose versus standard dose (back-to-back) adrenocorticotrophic stimulation tests in children and young adults with thalassemia major. Indian J Endocr Metab. 2013;17(6):1046–1052. 10.4103/2230-8210.122620 PMC3872683 24381882

[6] De Sanctis V, Soliman A, Candini G, et al. High prevalence of central hypothyroidism in adult patients with β-thalassemia major. Georgian Med News. 2013;6(222):88–94. 24099820

[7] De Sanctis V, Elsedfy H, Soliman AT, et al. Acquired hypogonadotropic hypogonadism (AHH) in thalassaemia major patients: an underdiagnosed condition? Mediterr J Hematol Infect Dis. 2016;8(1):e2016001. 26740862 10.4084/MJHID.2016.001 PMC4696472

[8] Bukhari SS, Junaid M, Rashid MU. Thalassemia, extramedullary hematopoiesis, and spinal cord compression: a case report. Surg Neurol Int. 2016;7(Suppl 5):S148–S152. 27069747 10.4103/2152-7806.177891 PMC4802988

[9] Korsten J, Grossman H, Winchester PH, et al. Extramedullary hematopoiesis in patients with thalassemia anemia. Radiology. 1970;95(2):257–263. 5439425 10.1148/95.2.257

[10] Taher A, Isma'eel H, Cappellini MD. Thalassemia intermedia: revisited. Blood Cells Mol Dis. 2006;37(1):12–20. 16737833 10.1016/j.bcmd.2006.04.005

[11] Munn RK, Kramer CA, Arnold SM. Spinal cord compression due to extramedullary hematopoiesis in beta-thalassemia intermedia. Int J Radiat Oncol Biol Phys. 1998;42(3):607–609. 9806521 10.1016/s0360-3016(98)00245-4

[12] Page MJ, McKenzie JE, Bossuyt PM, et al. The PRISMA 2020 statement: an updated guideline for reporting systematic reviews. BMJ. 2021;372:n71. 33782057 10.1136/bmj.n71 PMC8005924

[13] Institute JB. Checklist for case reports. The Joanna Briggs Institute critical appraisal tools for use in JBI systematic reviews; 2020. Available from: https://jbi.global/critical-appraisal-tools

[14] Ricchi P, Meloni A, Spasiano A, et al. Extramedullary hematopoiesis is associated with lower cardiac iron loading in chronically transfused thalassemia patients. Am J Hematol. 2015;90(11):1008–1012. 26228763 10.1002/ajh.24139

[15] Sousos N, Adamidou D, Klonizakis P, et al. Presence of the IVS-I-6-mutated allele in beta-thalassemia major patients correlates with extramedullary hematopoiesis incidence. Acta Haematol. 2017;137(3):175–182. 28399542 10.1159/000463919

[16] Chatterjee A, Sarkar S, Roy A. Haemothorax in thalassaemia may be a complication of rupture of intrathoracic extramedullary haematopoiesis. J Med Sci Clin Res. 2015;3(9): 7343-7346.

[17] Khan AM, Al-Sulaiti AM, Younes S, et al. The spectrum of beta-thalassemia mutations in the 22 Arab countries: a systematic review. Expert Rev Hematol. 2021;14(1):109–122. 33317346 10.1080/17474086.2021.1860003

[18] Subahi EA, Abdelrazek M, Yassin MA. Spinal cord compression due to extramedullary hematopoiesis in patient with beta thalassemia major. Clin Case Rep. 2021;9(1):405–409. 33489190 10.1002/ccr3.3542 PMC7812994

[19] Ahmad RW, Okar LA, Elhiday A, et al. Low back pain in beta thalassemia major revealing sacral extramedullay hematopoeisis: a case report. Clin Case Rep. 2021;9(5):e04258. 34084519 10.1002/ccr3.4258 PMC8142798

[20] Clark CA, Worden CP, Thorp BD, et al. Extramedullary hematopoiesis in the sinonasal cavity: a case report and review of the literature. Allergy Rhinol. 2020;11:2152656720918874. 10.1177/2152656720918874 PMC7177988 32363047

[21] Lund RE, Aldridge NH. Computed tomography of intracranial extramedullary hematopoiesis. J Comput Assist Tomogr. 1984;8(4):788–790. 6736389 10.1097/00004728-198408000-00042

[22] Yang X, Chen D, Long H, et al. The mechanisms of pathological extramedullary hematopoiesis in diseases. Cell Mol Life Sci. 2020;77(14):2723–2738. 31974657 10.1007/s00018-020-03450-w PMC11104806

[23] Fernández-García V, González-Ramos S, Martín-Sanz P, et al. Contribution of extramedullary hematopoiesis to atherosclerosis. The spleen as a neglected hub of inflammatory cells. Front Immunol. 2020;11:586527. 33193412 10.3389/fimmu.2020.586527 PMC7649205

[24] Haidar R, Mhaidli H, Taher AT. Paraspinal extramedullary hematopoiesis in patients with thalassemia intermedia. Eur Spine J. 2010;19(6):871–878. 20204423 10.1007/s00586-010-1357-2 PMC2899982

[25] Huang Y, Liu R, Wei X, et al. Erythropoiesis and iron homeostasis in non-transfusion-dependent thalassemia patients with extramedullary hematopoiesis. Biomed Res Int. 2019;2019:4504302. 30834265 10.1155/2019/4504302 PMC6374788

[26] Lanigan A, Fordham MT. Temporal bone extramedullary hematopoiesis as a cause of pediatric bilateral conductive hearing loss: case report and review of the literature. Int J Pediatr Otorhinolaryngol. 2017;97:135–138. 28483223 10.1016/j.ijporl.2017.03.032

[27] Sirisena M, Birman CS, McKibbin AJ, et al. Bilateral auditory ossicular expansions in a child with beta-thalassemia major: case report and literature review. Int J Pediatr Otorhinolaryngol. 2018;112:126–131. 30055721 10.1016/j.ijporl.2018.06.046

[28] Karimi M, Zarei T, Pishdad P. Extramedullary hematopoiesis in a patient with transfusion dependent beta-thalassemia presenting with cord compression. Iran J Blood Cancer. 2018;10(1):28–30.

[29] Shakeri R, Rahmati A, Zamani F. Photoclinic. Archiv Iran Med. 2013;16(5):315. 23641750

[30] Sumana G, Sanjay N, Habibul I. A rare case report of extra medullary hematopoiesis in lung in a case of thalassemia. Int J Health Res Med Legal Pract. 2014;2(2):134–137.

[31] Daneshbod Y, Kazemi T. Nodal extramedullary hematopoiesis and facial bone change in thalassemia. Blood. 2015;126(17):2070. 26788567 10.1182/blood-2015-07-652396

[32] Eskazan AE, Ar MC, Baslar Z. Intracranial extramedullary hematopoiesis in patients with thalassemia: a case report and review of the literature. Transfusion. 2012;52(8):1715–1720. 22220514 10.1111/j.1537-2995.2011.03499.x

[33] Aarabi B, Haghshenas M, Rakeii V. Visual failure caused by suprasellar extramedullary hematopoiesis in beta thalassemia: case report. Neurosurgery. 1998;42(4):922–925; discussion 925–926. 9574659 10.1097/00006123-199804000-00134

[34] A. Al-Namil S, Ma. Al-Diab J, N. Abdulnabi A. Adrenal extramedullary hematopoiesis in a patient with beta thalassemia major. MJBU. 2018;36(1):45–48.

[35] Nagaraj T, N U, Devarhubli AR, et al. B thalassemia major: a case report. J Int Oral Health. 2011;3(5):67–73.

[36] Tsitsikas DA, Barroso FA, Telfer P, et al. A patient with beta thalassaemia major and back pain. BMJ. 2008;337:a2304. 19019876 10.1136/bmj.a2304

[37] Wanchaitanawong W, Tantiworawit A, Piriyakhuntorn P, et al. The association between pre-transfusion hemoglobin levels and thalassemia complications. Hematology. 2021;26(1):1–8. 33357151 10.1080/16078454.2020.1856513

[38] Atmakusuma TD, Saragih EYP, Rajabto W. Achievement of pre-and post-transfusion hemoglobin levels in adult transfusion-dependent beta thalassemia: associated factors and relationship to reduction of spleen enlargement. IJGM. 2021;14:7515–7521. 10.2147/IJGM.S338114 PMC8570285 34754224

[39] Brewin J, El Hoss S, Strouboulis J, et al. A novel index to evaluate ineffective erythropoiesis in hematological diseases offers insights into sickle cell disease. Haematologica. 2022;107(1):338–341. 34670360 10.3324/haematol.2021.279623 PMC8719095

[40] Kopp CR, Kumar M, Sandal R, et al. Thalasaaemia and extramedullary haematopoiesis. QJM. 2019;112(7):543–544. 30629265 10.1093/qjmed/hcz008

[41] Doctor PN, Merchant R, Ak P. Paraspinal extramedullary hematopoiesis in beta thalassemia intermedia treated with low dose radiotherapy. Pediatrics. 2020;146(1_MeetingAbstract):452–454.

[42] Ahmad R, Okar L, Almasri H, et al. Low back pain in beta thalassemia major revealing sacral extra medullay hematopoeisis: a case report. Clin Case Rep. 2021;9:e04258. 10.1002/ccr3.4258 PMC8142798 34084519

