# Supplementary material for: Extramedullary haematopoiesis in patients with transfusion dependent β-thalassaemia (TDT): a systematic review
Source: Ann Med. 2022 Mar 9;54(1):764–74. doi: 10.1080/07853890.2022.2048065 (PMC8941948; doi:10.1080/07853890.2022.2048065)
Supplement: Supplemental Material [file IANN_A_2048065_SM7678.pdf]

Articles included in the systematic review for EMH in the context of TDT[1-72]

1. A. Al-Namil S, MA. Al-Diab J, N. Abdulnabi A. Adrenal Extramedullary Hematopoiesis in a patient with Beta Thalassemia Major. The Medical Journal of Basrah University. 2018;36(1):45-8. doi: 10.33762/mjbu.2018.145198.
2. Aarabi B, Haghshenas M, Rakeii V. Visual failure caused by suprasellar extramedullary hematopoiesis in beta thalassemia: case report. Neurosurgery. 1998;42(4):922-5; discussion 5-6. Epub 1998/05/09. doi: 10.1097/00006123-199804000-00134. PubMed PMID: 9574659.
3. Abir K, Yousser M, Omar D, Alcheikh PS. Unusual Pulmonary Masses in Beta Thalassemia Major. EC PULMONOLOGY AND RESPIRATORY MEDICINE. 2016;2(5):181-6.
4. Ahad Aziz Qureshi PA, Razi M, Rehman A, Hassan A. Hodgkin lymphoma with co-existing extramedullary haematopoiesis in a Thalasaemia Major patient: Killing two birds with one stone using PET-CT. J Pak Med Assoc. 2019;69(7):1059. Epub 2020/01/28. PubMed PMID: 31983750.
5. Ahmad R, Okar L, Almasri H, Eldean MS, Elhiday A, Ata F, et al. LOW BACK PAIN IN BETA THALASSEMIA MAJOR REVEALING SACRAL EXTRA MEDULLAY HEMATOPOEISIS: A CASE REPORT. Authorea Preprints. 2020.
6. Al-Aabassi A, Murad BA. Presacral extramedullary hematopoiesis: a diagnostic confusion concerning a rare presentation. Med Princ Pract. 2005;14(5):358-62. Epub 2005/08/17. doi: 10.1159/000086936. PubMed PMID: 16103704.
7. Aliberti B, Patrikiou A, Terentiou A, Frangatou S, Papadimitriou A. Spinal cord compression due to extramedullary haematopoiesis in two patients with thalassaemia: complete regression with blood transfusion therapy. Journal of Neurology. 2001;248(1):18-22. doi: 10.1007/s004150170264.
8. Ben Ammar L, Ferjani H, Maatallah K, Bouallegue S, Riahi H, Kaffel D, et al. Spinal cord compression by extramedullary hematopoiesis in beta-thalassemia major. Clin Case Rep. 2020;8(8):1433-6. Epub 2020/09/05. doi: 10.1002/ccr3.2924. PubMed PMID: 32884769; PubMed Central PMCID: PMCPMC7455443.
9. Borgna-Pignatti C, Turlá G, Zanforlin N, Marsella M, Gentile MP, Maida G, et al. A calcified mass in the spinal canal of a patient with β-thalassemia major. J Pediatr Hematol Oncol. 2010;32(8):621. Epub 2010/09/08. doi: 10.1097/MPH.0b013e3181e5e221. PubMed PMID: 20818273.
10. Boussaa H, Kaffel D, Maatallah K, Lassoued Ferjani H, Hamdi W. A rare cause of lumbar nerve root compression: Extramedullary hematopoiesis in a patient with thalassemia major. Clinical Case Reports. n/a(n/a):e04119. doi: https://doi.org/10.1002/ccr3.4119.
11. Boyacigil S, Ali A, Ardiç S, Yüksel E. Epidural extramedullary haemopoiesis in thalassaemia. Australas Radiol. 2002;46(2):180-2. Epub 2002/06/13. doi: 10.1046/j.1440-1673.2001.01031.x. PubMed PMID: 12060158.
12. Bronn LJ, Paquelet, Tetalman MR. Intrathoracic extramedullary hematopoiesis: appearance on 99mTc sulfur colloid marrow scan. American Journal of Roentgenology. 1980;134(6):1254-5. doi: 10.2214/ajr.134.6.1254.
13. Bruneteau G, Fénelon G, Khalil A, Kanfer A, Girot R. [Spinal cord compression secondary to extramedullary hematopoiesis in a patient with thalassemia]. Rev Neurol (Paris). 2000;156(5):510-3. Epub 2000/06/09. PubMed PMID: 10844371.
14. Campisi S, Mangiagli A, De Sanctis V, Giovannini M. Paraplegia in a thalassaemic patient with short stature. Pediatr Endocrinol Rev. 2011;8 Suppl 2:340-4. Epub 2011/06/28. PubMed PMID: 21705991.

15. Chatterjee A, Sarkar S, Roy A. Haemothorax in Thalassaemia May be a Complication of Rupture of Intrathoracic Extramedullary Haematopoiesis. 2015.
16. Chen Y-W, Sheu R-S, Chiou S-S, Chang T-T, Huang Y-F, Liu G-C. Tc99m sulfur colloid scintigraphy for detection of intrathoracic extramedullary hematopoiesis in patient with beta-thalassemia major--a case report. The Kaohsiung journal of medical sciences. 2000;16(6):319-24.
17. Chiam QL, Lau KK. Extramedullary haematopoiesis in thalassaemia major causing spinal cord compression. Australas Radiol. 2007;51(2):168-71. Epub 2007/04/11. doi: 10.1111/j.1440-1673.2007.01689.x. PubMed PMID: 17419864.
18. Chuang CK, Chu SH, Fang JT, Wu JH. Adrenal extramedullary hematopoietic tumor in a patient with beta-thalassemia. J Formos Med Assoc. 1998;97(6):431-3. PubMed PMID: 9650475.
19. Daneshbod Y, Kazemi T. Nodal extramedullary hematopoiesis and facial bone change in thalassemia. Blood. 2015;126(17):2070. Epub 2016/01/21. doi: 10.1182/blood-2015-07-652396. PubMed PMID: 26788567.
20. Diaconu A, Coculescu BI, Rizea O, Herlea V, Vultur H. Extramedullary hematopoiesis in beta thalassemia major-case presentation. Romanian Journal of Legal Medicine. 2020;28(2):212-7. doi: 10.4323/rjlm.2020.212.
21. Dragean CA, Duquesne L, Theate I, Ghaye B, Coche EE. Extramedullary haemopoiesis and spinal cord compression. The Lancet. 2011;377(9761):251. doi: 10.1016/S0140-6736(10)60485-0.
22. Elbers H, Stadt Jvd, Wagenaar SS. Tumor-Simulating Thoracic Extramedullary Hematopoiesis. The Annals of Thoracic Surgery. 1980;30(6):584-7. doi: https://doi.org/10.1016/S0003-4975(10)61735-3.
23. Emamhadi M, Alizadeh A. Effect of hypertransfusion on extramedullary hematopoietic compression mass in thalassemia major: a case report. Iran J Radiol. 2012;9(3):154-6. Epub 2013/01/19. doi: 10.5812/iranjradiol.8064. PubMed PMID: 23329982; PubMed Central PMCID: PMCPMC3522378.
24. Eskazan AE, Ar MC, Baslar Z. Intracranial extramedullary hematopoiesis in patients with thalassemia: a case report and review of the literature. Transfusion. 2012;52(8):1715-20. Epub 2012/01/10. doi: 10.1111/j.1537-2995.2011.03499.x. PubMed PMID: 22220514.
25. Fareed S, Soliman AT, De Sanctis V, Kohla S, Soliman D, Khirfan D, et al. Spinal cord compression secondary to extramedullary hematopoiesis: A rareness in a young adult with thalassemia major. Acta Biomed. 2017;88(2):237-42. Epub 2017/08/29. doi: 10.23750/abm.v88i2.6221. PubMed PMID: 28845843; PubMed Central PMCID: PMCPMC6166144.
26. Gaduputi V, Loganathan J, Nayudu SK, Chilimuri S. Beta-Thalassemia Presenting as an Acute Neurological Complication: A Case Report2011.
27. Kaitoukov Y, Zdanovich E, Moser T. Extramedullary Hematopoiesis in Beta Thalassemia Major: Multisystem Involvement. OMICS J Radiol. 2016;5:234.
28. Karaca F, Usul Afsar C, Sert F, Guner S, Ercolak V, Erkurt E, et al. Palliative Radiotherapy for Spinal Extramedullary Hematopoiesis in Thalassemia Major. International Blood Research & Reviews. 2015;3. doi: 10.9734/IBRR/2015/17235.
29. Karami H, Kosaryan M, Taghipour M, Sharifian R, Aliasgharian A, Motalebi M. Extramedullary hematopoiesis presenting as a right adrenal mass in a patient with Beta thalassemia. Nephrourol Mon. 2014;6(5):e19465. Epub 2015/02/20. doi: 10.5812/numonthly.19465. PubMed PMID: 25695031; PubMed Central PMCID: PMCPMC4318012.
30. Karimi M, Cohan N, Bagheri MH, Lotfi M, Omidvari S, Geramizadeh B. A lump on the head. The Lancet. 2008;372(9647):1436. doi: 10.1016/S0140-6736(08)61590-1.
31. Karimi M, Cohan N, Pishdad P. Hydroxyurea as a first-line treatment of extramedullary hematopoiesis in patients with beta thalassemia: Four case reports. Hematology. 2015;20(1):53-7. Epub 2014/04/11. doi: 10.1179/1607845414y.0000000168. PubMed PMID: 24717020.

32. Karimi M, Zarei T, Pishdad P. Extramedullary hematopoiesis in a patient with blood transfusion-dependent beta-thalassemia presenting with a mass like lesion and cord compression. Iranian Journal of Blood & Cancer. 2017.
33. Karki B, Xu YK, Tamrakar K, Wu YK. Intracranial extramedullary hematopoiesis in beta-thalassemia. Korean J Radiol. 2012;13(2):240-3. Epub 2012/03/23. doi: 10.3348/kjr.2012.13.2.240. PubMed PMID: 22438693; PubMed Central PMCID: PMCPMC3303909.
34. Keikhaei B, Shirazi AS, Pour MM. Adrenal extramedullary hematopoiesis associated with β-thalassemia major. Hematol Rep. 2012;4(2):e7. Epub 2012/07/25. doi: 10.4081/hr.2012.e7. PubMed PMID: 22826797; PubMed Central PMCID: PMCPMC3401136.
35. Khurana R, Arora SK, Hemal A, Arora S. Successful non-operative management of cauda equina syndrome in a case of thalassemia major. Pediatric Hematology Oncology Journal. 2016;1(2):41-3. doi: https://doi.org/10.1016/j.phoj.2016.07.004.
36. Konstantopoulos K, Vagiopoulos G, Kantouni R, Lymperi S, Patriarcheas G, Georgakopoulos D, et al. A case of spinal cord compression by extramedullary haemopoiesis in a thalassaemic patient: a putative role for hydroxyurea? Haematologica. 1992;77(4):352-4. Epub 1992/07/01. PubMed PMID: 1385278.
37. Kopparthy P, Kelkar AH, Aggarwal K, De Filippis S, Fletcher B. Red Blood Cell Exchange in a Patient With Extramedullary Hematopoiesis and Cor Pulmonale Secondary to Beta Thalassemia. Cureus. 2021;13(3):e13638. Epub 2021/04/08. doi: 10.7759/cureus.13638. PubMed PMID: 33824791; PubMed Central PMCID: PMCPMC8011980.
38. Lanigan A, Fordham MT. Temporal bone extramedullary hematopoiesis as a causeof pediatric bilateral conductive hearing loss:Case report and review of the literature. Int J Pediatr Otorhinolaryngol. 2017;97:135-8. Epub 2017/05/10. doi: 10.1016/j.ijporl.2017.03.032. PubMed PMID: 28483223.
39. Lau SK, Chan CK, Chow YY. Cord compression due to extramedullary hemopoiesis in a patient with thalassemia. Spine (Phila Pa 1976). 1994;19(21):2467-70. Epub 1994/11/01. doi: 10.1097/00007632-199411000-00019. PubMed PMID: 7846603.
40. Malinovská V, D'Andrea V, Dítě P, Malinovský L, Baselice PF, Bartolucci R, et al. [Hemothorax in the posterior mediastinum in extramedullary hemopoiesis]. Vnitr Lek. 1996;42(8):555-6. Epub 1996/08/01. PubMed PMID: 8967026.
41. Nagaraj T, N U, Devarhubli AR, SN S. B Thalassemia major: A case

report. J Int Oral Health 2011;3(5):67-73.
42. Nair R, Anand D, Menon G, Kiran Acharya K, Shastri B. Compressive myelopathy - An unusual presentation of extramedullary hematopoiesis in a known patient of thalassemia major. Archives of Medicine and Health Sciences. 2020;8(1):96-9. doi: 10.4103/amhs.amhs_97_20.
43. Oermann EK, Coppa ND, Margolis M, Sandhu FA. Extramedullary hematopoietic tumor mimicking a thoracic nerve root schwannoma. J Neurosurg Spine. 2010;13(1):78-81. Epub 2010/07/03. doi: 10.3171/2010.3.Spine09277. PubMed PMID: 20594021.
44. Oğüş C, Ozdemir T, Kabaalioğlu A. Right hilar mass in a patient with beta-thalassemia major. Respiration. 2001;68(2):215-6. Epub 2001/04/05. doi: 10.1159/000050496. PubMed PMID: 11287840.
45. Özer H, Kocaman Ü, Yılmaz M, Dalbasti T. Paraparesis Due to a Long-segment Thoracic Mass in a Beta Thalassemia Major Patient: Approach to the Treatment. Journal of Neurological Sciences. 2015;32:199-202.
46. Qiu D, Hu X, Xu L, Guo X. Extramedullary hematopoiesis on 18F-FDG PET/CT in a patient with thalassemia and nasopharyngeal carcinoma: A case report and literature review. J Cancer Res Ther. 2015;11(4):1034. Epub 2016/02/18. doi: 10.4103/0973-1482.150359. PubMed PMID: 26881631.
47. Rasekhi AR, Sh O, Rasekhi AR. Cord compression secondary to epidural extramedullary hematopoiesis in beta-thalassemia. Irn J Med Sci. 1998;23:138-41.

48. Reames DL, Lindstrom K, Raghavan P, Jane J. Extramedullary hematopoiesis within the clivus: an unusual cause of lower cranial nerve palsy. Journal of Neuro-Oncology. 2010;100(3):481-5. doi: 10.1007/s11060-010-0208-7.
49. Reif J, Graf N. Spinal space occupying lesions in thalassemia major. Neurosurgical Review. 1989;12(4):323-31. doi: 10.1007/BF01780851.
50. Ricchi P, Meloni A, Spasiano A, Neri MG, Gamberini MR, Cuccia L, et al. Extramedullary hematopoiesis is associated with lower cardiac iron loading in chronically transfused thalassemia patients. American Journal of Hematology. 2015;90(11):1008-12. doi: 10.1002/ajh.24139.
51. Richter E. [Extramedullary hematopoiesis with intraspinal extension in thalassemia]. Aktuelle Radiol. 1993;3(5):320-2. PubMed PMID: 8399424.
52. Saha A, Chattopadhyay S, Azam M, Chatterjee K. Thoracic spinal cord compression due to xtramedullary haemopoiesis in a patient with beta-thalassemia: complete clinical regression with radiation therapy alone. International Journal of Cancer Therapy and Oncology. 2014;3:03017. doi: 10.14319/ijcto.0301.7.
53. Salehi SA, Koski T, Ondra SL. Spinal cord compression in beta-thalassemia: case report and review of the literature. Spinal Cord. 2004;42(2):117-23. Epub 2004/02/07. doi: 10.1038/sj.sc.3101544. PubMed PMID: 14765145.
54. Sanei Taheri M, Birang SH, Shahnazi M, Hemadi H. Large Splenic Mass of Extramedullary Hematopoiesis. Iranian Journal of Radiology. 2005;2:98-101.
55. Sekar S, Burad D, Abraham A, Paul MJ. Adrenal incidentaloma caused by extramedullary haematopoiesis: conservative management is optimal. BMJ Case Rep. 2015;2015. Epub 2015/09/24. doi: 10.1136/bcr-2015-211014. PubMed PMID: 26392447; PubMed Central PMCID: PMCPMC4577657.
56. Shakeri R, Rahmati A, Zamani F. Photoclinic. Archives of Iranian medicine. 2013;16(5):315.
57. Sirisena M, Birman CS, McKibbin AJ, O'Brien KJ. Bilateral auditory ossicular expansions in a child with beta-thalassemia major: Case report and literature review. Int J Pediatr Otorhinolaryngol. 2018;112:126-31. Epub 2018/07/30. doi: 10.1016/j.ijporl.2018.06.046. PubMed PMID: 30055721.
58. Soman S, Rosenfeld DL, Roychowdhury S, Drachtman RA, Cohler A. Cord Compression due to Extramedullary Hematopoiesis in an Adolescent with Known Beta Thalassemia Major. J Radiol Case Rep. 2009;3(1):17-22. Epub 2009/01/01. doi: 10.3941/jrcr.v3i1.83. PubMed PMID: 22470615; PubMed Central PMCID: PMCPMC3303263.
59. Sousos N, Adamidou D, Klonizakis P, Agapidou A, Theodoridou S, Spanos G, et al. Presence of the IVS-I-6-Mutated Allele in Beta-Thalassemia Major Patients Correlates with Extramedullary Hematopoiesis Incidence. Acta Haematol. 2017;137(3):175-82. Epub 2017/04/12. doi: 10.1159/000463919. PubMed PMID: 28399542.
60. Subahi EA, Abdelrazek M, Yassin MA. Spinal cord compression due to extramedullary hematopoiesis in patient with Beta thalassemia major. Clin Case Rep. 2021;9(1):405-9. Epub 2021/01/26. doi: 10.1002/ccr3.3542. PubMed PMID: 33489190; PubMed Central PMCID: PMCPMC7812994.
61. Sumana G, Sanjay N, Habibul I. A Rare Case Report of Extra Medullary Hematopoiesis in Lung in a Case of Thalassemia. 2014.
62. Suresh SC, Raju B, Jumah F, Nanda A. Lumbosacral extradural extramedullary hematopoiesis in thalassemia major causing spinal canal stenosis. Surg Neurol Int. 2020;11:331. Epub 2020/11/17. doi: 10.25259/sni_563_2020. PubMed PMID: 33194265; PubMed Central PMCID: PMCPMC7656047.
63. Tai SM, Chan JS, Ha SY, Young BW, Chan MS. Successful treatment of spinal cord compression secondary to extramedullary hematopoietic mass by hypertransfusion in a patient with thalassemia major. Pediatr Hematol Oncol. 2006;23(4):317-21. Epub 2006/04/20. doi: 10.1080/08880010600629676. PubMed PMID: 16621773.

64. Talapatra K, Nemade B, Siddha M, Muckaden M, Laskar S. Extramedullary haematopoiesis causing spinal cord compression: A rare presentation with excellent outcome. Annals of Indian Academy of Neurology. 2007;10. doi: 10.4103/0972-2327.33221.
65. Tiwari A, Varghese V, Chand A, Chhabra M. Paraplegia due to Extramedullary Hematopoietic Tissue Compression of Thoracic Thecal Sac in a Patient with Thalassemia Treated Successfully with Surgical Decompression: A Case Report. Indian Journal of Neurosurgery. 2016;05. doi: 10.1055/s-0036-1572382.
66. Tsitsikas DA, Barroso FA, Telfer P, Kaya B, Evanson J, Provan A. A patient with beta thalassaemia major and back pain. Bmj. 2008;337:a2304. Epub 2008/11/21. doi: 10.1136/bmj.a2304. PubMed PMID: 19019876.
67. Usmani S, Ahmed N, Muzaffar S, Al Kandari F. Extensive Extramedullary Hematopoiesis in Thalassemia: Soft Tissue Uptake on 99mTc-MDP SPECT/CT. Clin Nucl Med. 2020;45(10):e459-e60. Epub 2020/07/14. doi: 10.1097/rlu.0000000000003191. PubMed PMID: 32657877.
68. Valotassiou V, Angelidis G, Alexiou S, Psimadas D, Tsougos I, Georgoulias P. Detection of Extramedullary Hematopoietic Tissue in a Patient with Beta-Thalassemia Major on Tc99m-Sestamibi Parathyroid Scintigraphy. Indian J Nucl Med. 2019;34(4):324-5. Epub 2019/10/04. doi: 10.4103/ijnm.IJNM_115_19. PubMed PMID: 31579358; PubMed Central PMCID: PMCPMC6771193.
69. Varshney A, Barwa V, Saini L, Lamba P, Yadav RK. Intracranial extramedullary hematopoiesis in a thalassemic girl: A Case report. Indian Journal of Case Reports. 2017;3(2):81-4. doi: 10.32677/IJCR.2017.v03.i02.010.
70. White K, Blay C, Ataya A, Alnuaimat H, Reddy R. A 34-Year-Old Man With Bilateral Paraspinal Masses and Shortness of Breath. Chest. 2019;156(5):e99-e102. Epub 2019/11/09. doi: 10.1016/j.chest.2019.05.043. PubMed PMID: 31699236.
71. Zeighami S, Eslahi SA, Hosseini MM, Ariafar A, Pakbaz S, Rastegari M, et al. Extramedullary Hematopoiesis in a Man With β-Thalassemia: An Uncommon Cause of an Adrenal Mass. Annals of Colorectal Research. 2015;3(2):0-. doi: 10.17795/acr-29003.
72. Ziegler L, Lange M, Feiden W, Vogl T. Spinal cord compression in thalassemia major: value of MR imaging. European Radiology. 1991;1(1):81-4. doi: 10.1007/BF00540112.
